# Fibroblast Growth Factor Receptor 3 Amplified Metastatic Melanoma Treated With Erdafitinib

**DOI:** 10.7759/cureus.11231

**Published:** 2020-10-29

**Authors:** Saro Sarkisian, Alyson McIntosh, Suresh Nair, Alexander N Shoushtari, Margaret Callahan

**Affiliations:** 1 Hematology and Oncology, Lehigh Valley Cancer Institute, Allentown, USA; 2 Radiation Oncology, Lehigh Valley Cancer Institute, Allentown, USA; 3 Hematology/Oncology, Lehigh Valley Cancer Institute, Allentown, USA; 4 Medicine, Melanoma Service, Memorial Sloan Kettering Cancer Center, New York City, USA; 5 Hematology and Oncology, Memorial Sloan Kettering Cancer Center, New York City, USA

**Keywords:** diffuse metastatic melanoma, immunotherapy, targeted therapy

## Abstract

The treatment of metastatic melanoma has changed dramatically in the last decade with the introduction of immunotherapy and targeted therapy. A futile disease in the past is now treated with various options, resulting in improvement in progression-free and overall survivals, along with improvement in the quality of life. Having said that, the majority of patients with metastatic melanoma eventually succumb to the disease. Molecular profiling of each tumor in the advanced stage is standard of care now, as this would lead to individualized treatment options for each patient. Here, we present a rare case of fibroblast growth factor receptor 3 (FGFR 3) amplified metastatic melanoma, treated rather unconventionally with FGFR 3 inhibitor erdafitinib.

## Introduction

Over the past two decades, the incidence of cutaneous melanoma has increased substantially, according to the Surveillance, Epidemiology and End Results database, from 38,000 in 1997 to 76,000 in 2016 [1]. Prior to the introduction of immunotherapy and targeted therapy, metastatic melanoma was historically treated with chemotherapeutic agents, such as dacarbazine and its prodrug temozolomide. Platinum agents were also used, albeit less frequently. The outcomes during the era of chemotherapy in metastatic melanomas were poor and prognosis dismal. 

Tumor profiling led to substantial improvement in our understanding of the pathophysiology of advanced melanomas. In 40%-60% of advanced melanomas, activating mutations are identified in BRAF V600E [2-4]. This led to clinical trials directly inhibiting BRAF, along with other trials looking at combination BRAF/MEK inhibition, leading to significant improvement in overall survival. Fibroblast growth factor receptor 3 (FGFR 3) is mutated in approximately 3.2% of patients with melanoma [5]. In a study by Li et al., FGFR3 alteration was highly expressed in cutaneous metastatic melanomas, and associated with increased Breslow thickness and lymph node metastases [6]. It is also noted that FGFR3 alteration is seen more in metastatic melanoma cells than the primary tumor [7]. Overexpression of FGFR3 has also been associated with several types of cancer, including multiple myeloma, bladder cancer, non-small cell lung cancer oropharyngeal squamous cell carcinoma [8-11].

## Case presentation

A 29-year-old woman presented to our hospital with shortness of breath and back pain. CT scan revealed a large right-sided pleural effusion, right paraspinal 7 cm soft tissue mass, and pericardial and right tracheobronchial lymphadenopathy. She underwent thoracentesis at which time 2.5L bloody fluid was removed. Cytology was positive for malignancy. CT-guided biopsy of the soft tissue mass revealed melanoma. Positron emission tomography/computed tomography (PET/CT) revealed hypermetabolic lesions in the right pleura, extensive mediastinal lymphadenopathy, adrenal and peritoneal metastases with occult primary on dermatology exam. MRI of the brain was negative.

Tumor profiling revealed BRAF, KIT, NTRK1/2/3, N/K/HRAS wild type disease. Pathology was confirmed at Brigham and Women’s and Memorial Sloan Kettering. She was started on combination immunotherapy with ipilimumab 3mg/kg and nivolumab 1 mg/kg every 21 days. Her course was complicated by post-treatment pleuritic pain which required brief interruption of combination anti-CTLA4 and PD1 inhibitors and short course of prednisone.

**Figure 1:**
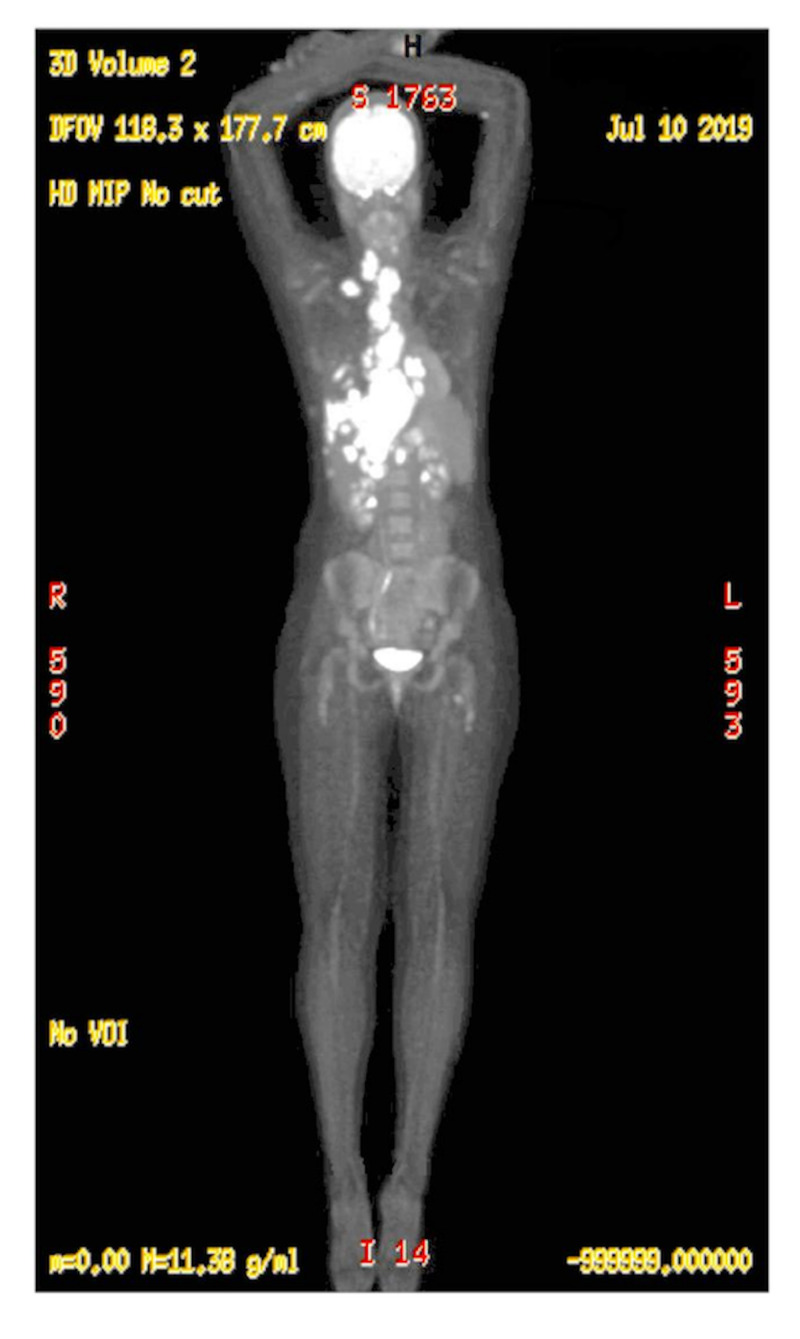
PET/CT prior to initiation of erdafitinib PET/CT, positron emission tomography/computed tomography

After four cycles of treatment, restaging PET/CT revealed worsening mediastinal and retroperitoneal adenopathy (Figure 1). MRI of the brain revealed 20 mostly sub-centimeter lesions suggesting metastases. She was given palliative carboplatin/paclitaxel and had gamma knife to 10 of the largest brain lesions. She only received one cycle of carboplatin/paclitaxel and had clinical progression with increased cervical lymphadenopathy and increased daily bloody pleural drainage, around 400cc per day. STRATA Oncology NexGenSequencing revealed FGFR3 amplification with an estimated copy number of nine. 

**Figure 2:**
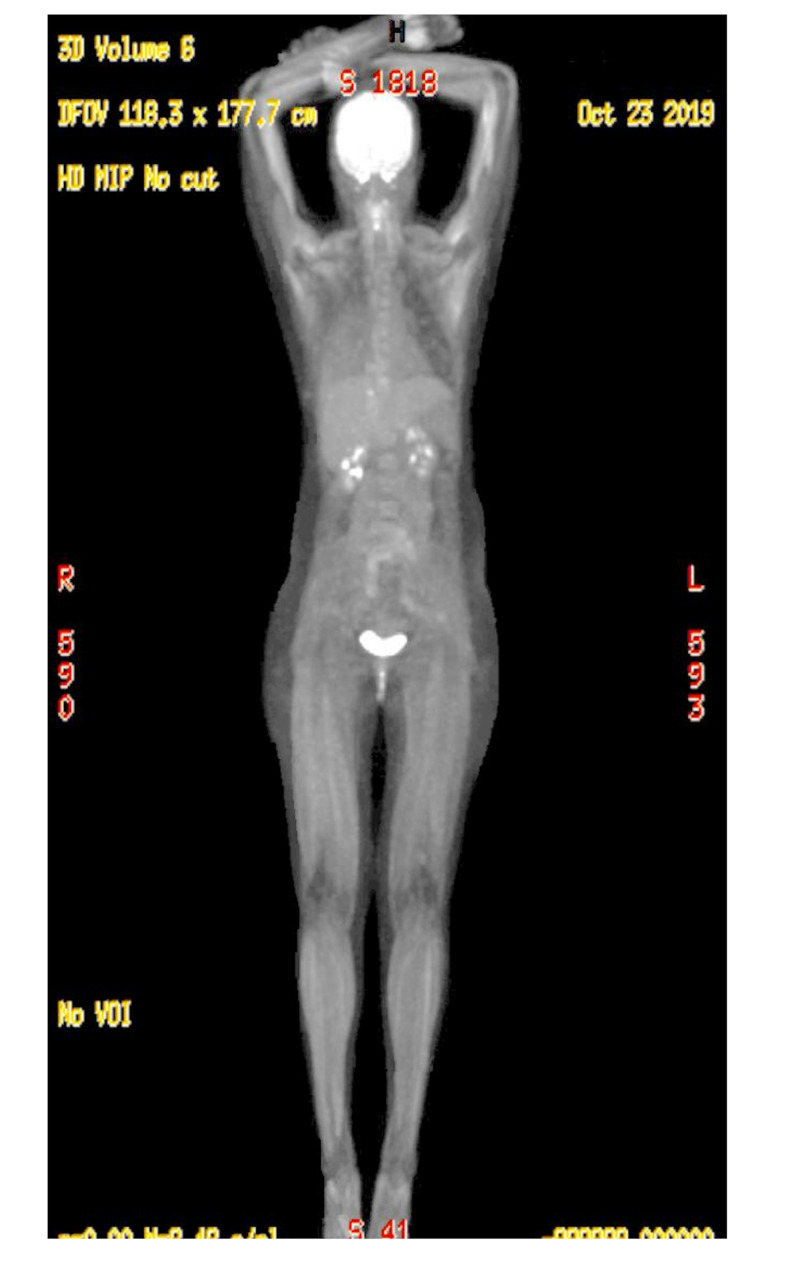
PET/CT after erdafitinib PET/CT, positron emission tomography/computed tomography

We obtained approval to use erdafitinib on a compassionate use program. This drug is approved for the treatment of locally advanced or metastatic urothelial carcinoma with susceptible FGFR3 or FGFR2 genetic alterations, that has progressed during or following platinum-containing chemotherapy, including within 12 months of neoadjuvant or adjuvant platinum-containing chemotherapy [12]. She tolerated erdafitinib 8 mg/day well and within three days, her drainage requirements decreased dramatically and her symptoms resolved. Restaging PET/CT was performed at 12 weeks showing a 95% resolution of systemic disease and metabolic uptake (Figure 2). MRI of the brain at six weeks showed overall stability of the brain lesions and dexamethasone was able to be weaned. At 16 weeks, extracranial disease was controlled, however, the patient developed seizures and progression in the brain. MRI brain then showed 40 metastatic lesions and leptomeningeal changes. She received whole-brain radiation therapy. Her overall course was complicated by worsening brain metastases and declining performance status. Repeat MRI brain revealed even more brain metastases resulting is significant morbidity, eventually the patient was transitioned to hospice care and passed away. 

## Discussion

Erdafitinib is a pan fibroblast growth factor receptor kinase inhibitor that was approved in locally advanced or metastatic urothelial carcinoma based on BCL2001 trial. This was an open label, phase 2 study that included a total of 99 patients with disease progression during or after at least one course of chemotherapy or within 12 months after neoadjuvant or adjuvant chemotherapy [13]. For this specific patient population, Erdafitinib led to median progression-free survival and overall survival of 5.5 months and 13.8 months, respectively. FGFR alteration is rare in melanoma. To our knowledge, this is the first case of FGFR 3-altered metastatic melanoma that was treated with Erdafitinib with significant response, albeit brief, after refractoriness to immunotherapy and chemotherapy. The discordant response in the brain and the rest of her body suggests either poor central nervous system (CNS) penetration of erdafitinib or more resistant mutations in the brain metastases. Our patient derived dramatic response and benefit with stabilization of extracranial disease from erdafitinib that lasted for approximately 27 weeks prior to progression. Clinical trials of FGFR 3 inhibitors in advanced melanoma driven by FGFR 3 alterations are warranted and better strategies for CNS disease control is needed. Through this personalized approach, we were able to prolong our patient's survival with improving her quality of life as well, however this short and substantial benefit would lead us to believe that further mutations or alterations have happened which we have not identified yet leading to this discordant response. 

## Conclusions

Immunotherapy and targeted therapy have improved the survival of patients with advanced melanomas. This case highlights that FGFR amplifications are a potentially relevant receptor tyrosine kinase in melanoma in general, and that FGFR2/3 inhibition could represent an additional target beyond BRAF/KIT/RAS/NTRK that could spur basket clinical trial participation. Through this case, we shed light on this rare clinical scenario with the hope of increasing awareness in helping other patients and the importance of personalized approach in caring for oncology patients in general.
